# Cardiomyocyte Protection by Hibernating Brown Bear Serum: Toward the Identification of New Protective Molecules Against Myocardial Infarction

**DOI:** 10.3389/fcvm.2021.687501

**Published:** 2021-07-16

**Authors:** Lucas Givre, Claire Crola Da Silva, Jon E. Swenson, Jon M. Arnemo, Guillemette Gauquelin-Koch, Fabrice Bertile, Etienne Lefai, Ludovic Gomez

**Affiliations:** ^1^Univ Lyon, CarMeN Laboratory, INSERM, INRA, INSA Lyon, Université Claude Bernard Lyon 1, Bron, France; ^2^Faculty of Environmental Sciences and Natural Resource Management, Norwegian University of Life Sciences, Ås, Norway; ^3^Department of Forestry and Wildlife Management, Inland Norway University of Applied Sciences, Koppang, Norway; ^4^Department of Wildlife, Fish, and Environmental Studies, Swedish University of Agricultural Sciences, Umeå, Sweden; ^5^Centre National d'Etudes Spatiales, CNES, Paris, France; ^6^University of Strasbourg, CNRS, IPHC UMR 7178, Laboratoire de Spectrométrie de Masse Bio-Organique, Strasbourg, France; ^7^Université Clermont Auvergne, INRAE, UNH, Clermont-Ferrand, France

**Keywords:** cardiomyocyte, hypoxia-reoxygenation injury, protection, bear serum, hibernation, novel therapeutic strategy

## Abstract

Ischemic heart disease remains one of the leading causes of death worldwide. Despite intensive research on the treatment of acute myocardial infarction, no effective therapy has shown clinical success. Therefore, novel therapeutic strategies are required to protect the heart from reperfusion injury. Interestingly, despite physical inactivity during hibernation, brown bears (*Ursus arctos*) cope with cardiovascular physiological conditions that would be detrimental to humans. We hypothesized that bear serum might contain circulating factors that could provide protection against cell injury. In this study, we sought to determine whether addition of bear serum might improve cardiomyocyte survival following hypoxia–reoxygenation. Isolated mouse cardiomyocytes underwent 45 min of hypoxia followed by reoxygenation. At the onset of reoxygenation, cells received fetal bovine serum (FBS; positive control), summer (SBS) or winter bear serum (WBS), or adult serums of other species, as indicated. After 2 h of reoxygenation, propidium iodide staining was used to evaluate cell viability by flow cytometry. Whereas, 0.5% SBS tended to decrease reperfusion injury, 0.5% WBS significantly reduced cell death, averaging 74.04 ± 7.06% vs. 79.20 ± 6.53% in the FBS group. This cardioprotective effect was lost at 0.1%, became toxic above 5%, and was specific to the bear. Our results showed that bear serum exerts a therapeutic effect with an efficacy threshold, an optimal dose, and a toxic effect on cardiomyocyte viability after hypoxia–reoxygenation. Therefore, the bear serum may be a potential source for identifying new therapeutic molecules to fight against myocardial reperfusion injury and cell death in general.

## Introduction

Despite significant advances in the ability to reperfuse ischemic myocardium and save heart tissue from reperfusion injury, ischemic heart disease remains one of the leading causes of death worldwide. Many therapeutic strategies have been studied, in particular methods of maintaining post-ischemic cell survival, the so-called cardioprotective interventions. However, although much has been learned about the methods and mechanisms of cardioprotection, no effective therapy has shown success in clinical translation.

Over the last decade of research in this area, most cardioprotective strategies have been designed to either target and inhibit a crucial cell death pathway or to activate a specific endogenous cardioprotective pathway (1, 2). We believe that the best strategy to improve both survival and quality of life in patients suffering from myocardial infarction is to minimize myocardial death that occurs due to reperfusion injury. It is also becoming clear that in addition to cardiomyocytes, cardioprotection should also target other cardiac or circulating cell types, and blood-cell-free circulating factors including globulins, micro-RNA, cytokines, receptors and adhesion molecules, which may provide direct or paracrine benefits. As such, there is a need to discover and investigate novel therapeutic targets for cardioprotection.

Many species of mammals, birds, and reptiles have evolved a strategy of reduced metabolic rate and energy conservation for prolonged periods by hibernating. During 4–6 months of hibernation, bears (*Ursus* spp.) do not eat, drink, or urinate; and they show minimal activity, yet they appear to retain normal organ function. Moreover, hibernating bears differ from hibernating rodents in that they maintain a higher body temperature [33–35°C (3) vs. <10°C], and they are reported to be shallow hibernators (4); however, they do not periodically arouse during the entire duration of their hibernation period. Such characteristics therefore make hibernating bears good models in a biomedical context.

To conserve energy during hibernation, the bear's oxygen demand is reduced to ~25% of the active state (5). Cardiovascular adaptations must occur for the myocardium to remain healthy and efficient during a period of extremely low heart rates and cardiac output (5–7). The cardiac adaptations during hibernation are characterized by a profound bradycardia with extreme respiration sinus arrhythmia and a preserved left ventricular ejection fraction, associated with a decrease in left ventricle mass/volume ratio indicating some degree of cardiac remodeling to adapt to the altered hemodynamic state (8–10). Interestingly, when they emerge from their dens in the spring, bears are free from cardiovascular diseases (11, 12), kidney failure (13–15), sarcopenia (16–18), osteoporosis (19, 20), and other deleterious conditions (21, 22). The contrast with physically inactive humans could not be greater (23–29).

Thus, the hibernation phenomenon is more than biologically interesting because understanding how organs cope with the stresses of hibernation could have direct clinical relevance (30) and especially for cardiovascular disease, such as myocardial infarction. Although we cannot rule out the possible role of parasympathetic and sympathetic nervous systems in the regulation of the cardiac function in bears entering or coming out of hibernation (3, 8), it is likely that circulating compounds may contribute to cardioprotection *in vivo*. Indeed, many blood components have already been proposed to be involved in a humoral mechanism of cardioprotection (31). Moreover, this hypothesis is reinforced by our recent demonstration that hibernating bear serum contains circulating components that can inhibit protein catabolism in cultured human muscle cells, with myosin accumulation in myotubes (32). Therefore, as a way to validate if hibernating bear serum actually contains circulating factors that could provide protection against cell death during hypoxia–reoxygenation (HR) injury, the objective of the present study was to evaluate the effectiveness of an acute treatment with bear serum at reoxygenation on the viability of post-ischemic primary mouse cardiomyocytes.

In this study, cardiomyocytes isolated from adult mice were exposed to HR sequence with brown bear (*Ursus arctos*) serum collected in winter and summer periods (WBS and SBS, respectively); and the therapeutic index of bear serum treatment was evaluated by flow cytometry. Our results showed that the addition of WBS was protective against reperfusion injury at the optimal dose of 0.5%. This effect was lost when the dose was reduced to 0.1% and became toxic above 5% of bear serum addition. Importantly, our results highlight that this profile effect seems to be specific to the serum from bear species.

Our study suggests that bear serum seems to be a potential source for identifying new therapeutic molecules to fight against human myocardial reperfusion injury and cell death in general.

## Materials and Methods

### Bear Serum

During the winters and summers of 2016 and 2019, blood samples were collected from the jugular vein of anesthetized free-ranging subadult (2- to 3-year-old) brown bears (nine females and four males) within 20 min after darting, as described previously (32, 33). Serum samples were prepared (3,000 g, 20 min) within 1 h after sampling and then stored at −80°C until the experiment. Both summer and winter mixes were obtained by pooling the same volume of serum for all bears [see ref. (32)].

### Hypoxia–Reoxygenation Model

As previously described (34, 35), adult cardiomyocytes were isolated from 8- to 12-week-old C57Bl/6J male and female mice (Charles River, L'Arbresle, France). Rod-shaped calcium-tolerant mouse cardiomyocytes were then subjected to a suspension-simulated hypoxia in a controlled hypoxic chamber (Eppendorf Galaxy 48R; Eppendorf, Hamburg, Germany), induced by nitrogen flushing up to 1% partial O_2_ pressure for 45 min, in 1 ml of a Tyrode solution (140 mM of NaCl, 5 mM of KCl, 10 mM of HEPES, 1 mM of MgCl_2_, and 1.8 mM of CaCl_2_ at pH 7.4 at 37°C) (36). Reoxygenation was induced at 37°C by the addition of 1 ml of normal culture medium [MEM #21575022 Gibco®, 10% fetal bovine serum (FBS), 10 mM of BDM, 100 U/ml of penicillin, 2 mM of glutamine, and 2 mM of ATP] supplemented with different serum concentrations. Control groups consisted of cell suspensions without hypoxic stress in a normal culture medium supplemented with different concentrations of each serum.

### Cell Death by Flow Cytometry

At the end of the HR protocol, cardiomyocytes were collected for flow cytometry analysis. Propidium iodide (PI; P4864 Sigma-Aldrich, St. Louis, MO, USA), a cell viability probe, was added extemporaneously before acquisition at 1 μg/ml. Flow cytometry experiments were conducted blindly using Fortessa X-20 (BD Biosciences, San Jose, CA, USA). In total, 1,000 events were acquired per tube. PI was excited at 561 nm, and the emission band-pass filter was collected at 620 nm. Cell death was represented by the percentage of positive cells for PI staining.

### Statistical Analysis

The data were analyzed with DIVA Software (BD Biosciences) and were quantified and expressed as mean ± SD, where indicated. Differences in means among multiple groups were analyzed using a two-way ANOVA followed by a Tukey's *post-hoc* test (two variables: experimental groups and experimental days). Statistical significance was set to a threshold of *p* ≤ 0.05. No data/animals were excluded from the study. Statistics were computed using GraphPad Prism 6.1 software (GraphPad Software, San Diego, CA, USA).

## Results

### Hypoxic Cardiomyocytes Treated With Bear Serum Exhibit Reduced Cell Death at Reoxygenation

**Figure 1:**
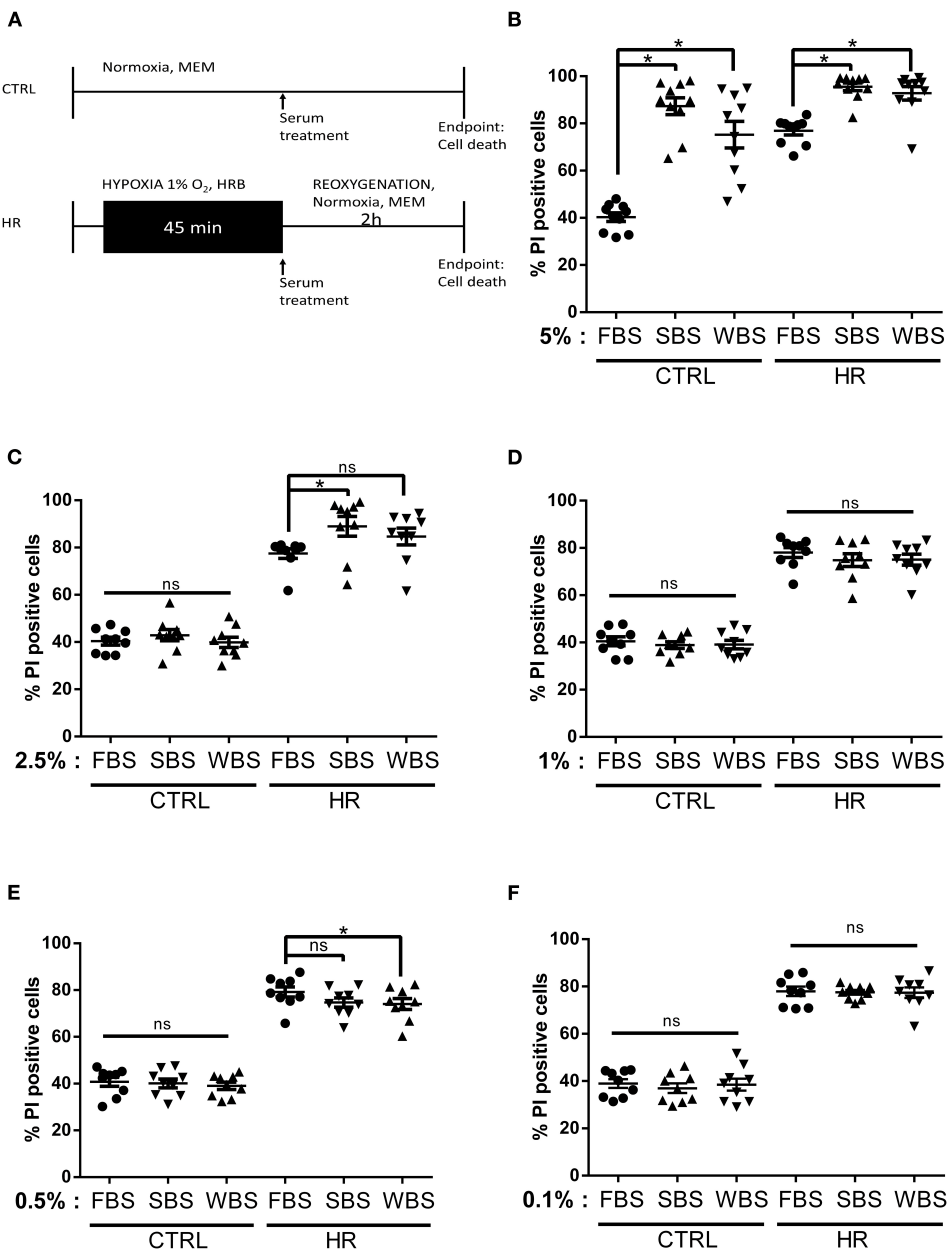
Dose–response effects of bear serum treatment on cardiomyocyte viability: **(A)** Experimental design. Isolated mouse cardiomyocytes underwent 45-min hypoxia followed by 2-h reoxygenation. At the onset of reoxygenation, normoxic and hypoxic cardiomyocytes received serum concentrations as indicated. The percentage of cell death was measured in both normoxic (CTRL) and hypoxic (HR) cells treated with **(B)** 5%, **(C)** 2.5%, **(D)** 1%, **(E)** 0.5%, and **(F)** 0.1% of fetal bovine serum (FBS), summer bear serum (SBS), and winter bear serum (WBS). Mean of propidium iodide (PI) positive cells ± SD, *n* = 9–10 different experimental days with 1,000 events/assay (*p < 0.05). Differences in means among multiple groups were analyzed using two-way ANOVA with a Tukey's *post-hoc* test (ns: non significant).

To determine whether bear serum might provide beneficial effects against reperfusion injury, we first mimicked the bear serum dose published by Chanon et al. (32). As shown in Figure 1A, control and hypoxic groups were supplemented at the onset of the reperfusion period with 5% of bear or FBS. Our results showed that, in control groups, the addition of 5% bear serum significantly increased cell death averaging 87.31 ± 11.31% and 75.23 ± 17.76% in SBS and WBS groups, respectively, as compared with 40.29 ± 5.77% in the FBS group (*p* < 0.05; Figure 1B). After HR, whereas cell death increased up to 76 ± 5.46% in the FBS group, most cardiomyocytes were dead in both SBS and WBS groups, reaching 95.50 ± 5.23% and 92.76 ± 9.01%, respectively. These surprising results suggest that bear serum treatment seems to be toxic for adult cardiomyocytes at a dose of 5%. Next, we chose to establish the dose–response effects of bear serum by reducing its concentration at reperfusion up to 0.1%.

Interestingly, at a dose of 2.5%, the toxicity of bear serum disappeared in control groups averaging 40.40 ± 4.9%, 42.91 ± 7.02%, and 39.90 ± 6.50% cell death in FBS, SBS (*p* = 0.958), and WBS (*p* > 0.999) groups, respectively (Figure 1C). However, after HR, although the cell death was not different between the WBS and FBS groups, averaging 84 ± 66% and 77.42% respectively, the addition of 2.5% of SBS still remained toxic, with a cell death averaging 88.96 ± 12.48% as compared with the FBS group (Figure 1C).

Our results showed that the addition of 1% bear serum provided no additional effect on cell death, neither in control groups, averaging 38.87 ± 4.17% in SBS (*p* = 0.97) and 40.48 ± 5.58% in WBS (*p* = 0.98) groups, nor in HR groups, averaging 74 ± 8.14% (*p* = 0.64) and 78.6.20% (*p* = 0.71), respectively, vs. FBS (Figure 1D). This suggests no advantage or disadvantage of bear serum treatment to the fate of cardiomyocytes in controls or after HR at this dose.

Treatment of control groups with 0.5% bear serum did not modify cell death, with 40 ± 5.56% in SBS group and 39.03 ± 4.68% in WBS vs. 40 ± 5.81% in the FBS group (*p* = ns; Figure 1E). On the other hand, whereas SBS tended to decrease cell death after HR (*p* = 0.09 vs. FBS), a treatment of 0.5% of WBS at reoxygenation significantly reduced cell death, averaging 74.04 ± 7.06% vs. 79.20 ± 6.53% in the FBS group (*p* < 0.05; Figure 1D). To rule out a possible imbalance in the protein content between summer and winter serums, we measured the total protein content of each serum mix. As reported in Supplementary Table 1, the total protein content was similar in each bear serum mix ranging from 15.95 ± 1.02 to 18.13 ± 1.29 mg/ml (*p* = ns). Bear serum collected during summer may nevertheless contain substances in concentrations that may provide protection against cell death and that this phenomenon is amplified with the serum collected in hibernating bears. By continuing to reduce the bear serum dose to 0.1%, our results showed no effect on survival of control cardiomyocytes, with death rates ranging from 36.97 ± 6.08% to 38.95 ± 7.60% (Figure 1F). The potential cardioprotective effect of bear serum was lost after HR with death rates of 77.54 ± 2.98% in the SBS group, 77.38 ± 6.68% in the WBS group, and 77.94 ± 5.98% in the FBS group (*p* > 0.99; Figure 1F).

Altogether, these results suggest that the efficiency of the bear serum against cell death was dependent on both the dose and the phenotype of sampled bears. Thus, our results highlight a potential cardioprotective role of WBS at 0.5%.

### The Therapeutic Effect of Serum Treatment on Cardiomyocyte Viability Is Specific of the Serum From Ursid Species

**Figure 2:**
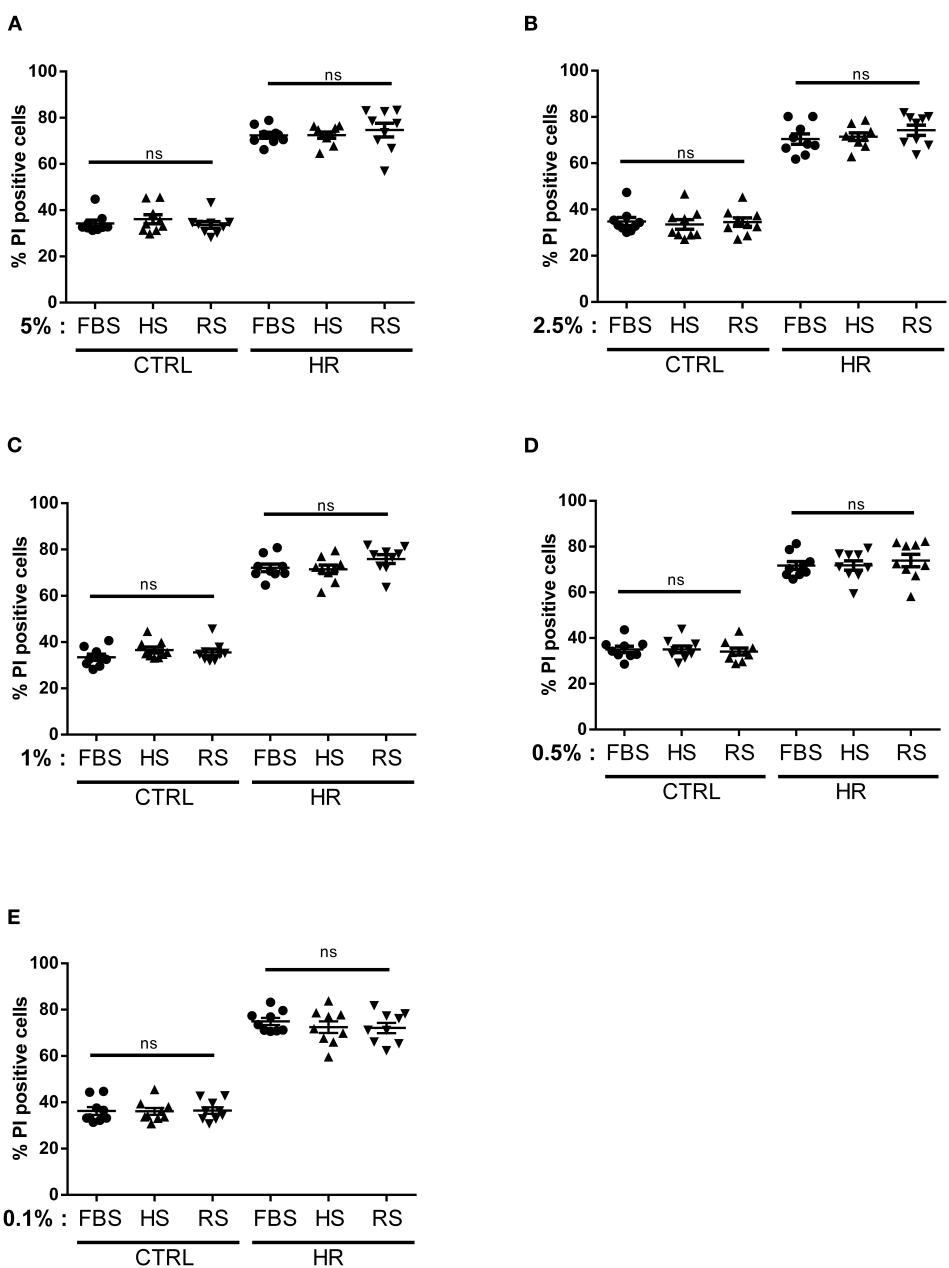
Dose–response effects of adult serum treatment on cardiomyocyte viability: percentage of cell death measured in both normoxic (CTRL) and hypoxic (HR) cells treated with **(A)** 5%, **(B)** 2.5%, **(C)** 1%, **(D)** 0.5%, and **(E)** 0.1% of fetal bovine serum (FBS), horse serum (HS), and/or rabbit serum (RS). Mean of propidium iodide (PI) positive cells ± SD, *n* = 9 different experimental days with 1,000 events/assay. Differences in means among multiple groups were analyzed using two-way ANOVA with a Tukey's *post-hoc* test (ns: non significant).

According to the literature, including cardiomyocyte isolation and maintenance (35, 36), FBS is the most used serum in cell culture. To check whether the serum from other animal species could exert the same effects, we repeated the previous experimental protocol with the addition of two different species of adult serum from horse (#H1138, Sigma) and rabbit (#R4505, Sigma). Similar to the bear samples, horse and rabbit adult serums were not decomplemented. Our results showed that the addition of horse or rabbit serum (5, 2.5, 1, 0.5, and 0.1%) did not influence cardiomyocyte viability in control conditions (Figure 2). Moreover, it is interesting to note that the absence of FBS also did not affect cell viability in control group averaging 34.09 ± 4.10%, ruling out the possibility that specific growth factors from the serum of subadult bears are involved in the observed effects. Altogether, these results show that, as was the case of fetal serum, the addition of adult horse or rabbit serum did not influence cell viability in our experimental conditions. Although HR stress significantly increased cell death in each HR groups (*p* < 0.05 vs. respecting control group), the addition of different concentrations of adult serums from horse and rabbit did not influence cell death rate (*p* = ns vs. HR FBS groups) (Figure 2). Altogether, these results suggested that the presence of horse or rabbit adult serum in the reoxygenation medium had no impact on the viability of cardiomyocytes after HR. Their use does not seem more advantageous than that of FBS. Moreover, without any dose–effect relationship of horse and rabbit adult serum on cell viability, our results did not show any threshold, toxic, or efficient effect on cell death. These results reinforced the conclusion that the efficacy profile we have observed in the presence of bear serum is specific to ursid species.

## Discussion

In this study, we described that bear serum presented a therapeutic profile from an efficacy threshold to a toxic effect on cardiomyocyte viability after HR. Although 0.5% SBS tended to decrease reperfusion injury, 0.5% WBS significantly reduced cell death after HR. This cardioprotective effect was lost at 0.1% and became adverse at 5%, which suggests a therapeutic window between 0.1 and 1% of bear serum. Moreover, our results demonstrated that this therapeutic profile was specific to the serum from brown bears, in contrast to horse or rabbit serum. As a new approach to overcome the lack of efficient treatment in clinical cardioprotection, our results suggest that hibernating bear serum might be a source for identifying new cardioprotective molecules.

Accordingly, these data reinforce previous research showing that treatment with serum could provide beneficial effects against some pathologies (37, 38). Indeed, it has been demonstrated that treatment of cells with serum from patients with myocardial infarction prevents inflammation in cardiomyocytes, thereby protecting healthy tissue (37). Others have demonstrated that human serum albumin treatment reduces ischemia–reperfusion injury in skeletal muscle in a rabbit model (38). Altogether, our results add to this that bear serum might contain molecules that confer cardioprotection against cell death during reperfusion injury.

Our data further demonstrated that, although SBS and WBS seem to induce similar responses to cardiomyocyte viability, WBS was more favorable than SBS. Indeed, when mimicking experimental protocols from our group on human myotubes, we were surprised to measure a toxic effect with 5% bear serum treatment, but it is worth noticing that the toxic effect was always lower in the hibernating serum, compared with the summer serum. According to the literature (39–45), the potential cytotoxicity of serum for *in vitro* cells culture involves mainly the activation of the complement, and several hormones and inhibitory growth factors, as well as the toxic effects of polyamines, exosomes, and potential molecules that cause oxidative stress and stimulate pro-inflammatory cytokine release known to trigger apoptosis. We cannot also exclude that high serum concentration may affect cell metabolism in our model. Such mechanisms remain to be investigated in depth in the future.

On the other hand, both serums tended to reduce cell death at reperfusion, but only WBS provided a significant reduction of 6% of cell death, compared with FBS positive controls. We are aware that this decrease is modest, but it is an encouraging result for the development of a new cardioprotection strategy. Moreover, it remains to be determined whether the promising effects that were observed may be affected by the anesthetic agents used to immobilize the bears. Now, we must optimize the protocol and the analysis to decipher the optimal dose between 0.1 and 1% serum treatment, the optimized temperature (because hibernating body bear temperature is reduced to 33–35°C), and to decipher which active molecules are similarly present in SBS and WBS and which molecules are not active to explain the efficacy gap between the seasons.

Several reports, including those of Kim et al. (46), provided the evidence that the composition of serum itself could influence myocardial mRNA, which may provide beneficial or deleterious effects in ischemia–reperfusion models. Among the circulating components that are known to show seasonal regulation in bears (47), fatty acids, whose composition are changed due to prolonged fasting, could play a role (32). Moreover, as described by Zolla (48), future studies are required to identify the therapeutic small molecules that confer cardioprotection *via* bear serum treatment. Finally, it is worth to notice that this novel therapeutic strategy was specific to the serum of Ursidae family origin, since supplementation with adult horse or rabbit serums did not impact cell viability as compared with respective controls.

## Conclusion

Our results demonstrate that although active molecules have not yet been identified, bear serum and more especially, hibernating bear serum provide specific cardioprotection against reperfusion injury. Our demonstration of the protective effect of serum molecules coming from hibernating animals in non-hibernating animals opens a new therapeutic avenue for identifying cardioprotective molecules with future applications in humans.

## Data Availability Statement

The original contributions presented in the study are included in the article/Supplementary Material, further inquiries can be directed to the corresponding author/s.

## Ethics Statement

The animal study was reviewed and approved by Swedish Ethical Committee on Animal Experiment (applications Dnr C3/2016 and Dnr C18/2015), the Swedish Environmental Protection Agency (NV-0741-18), the Swedish Board of Agriculture (Dnr 5.2.18–3060/17), and the French Claude Bernard Lyon 1 University animal research committees #19896-201903212127912.

## Author Contributions

EL, FB, and LGo: conceptualization and methodology. LGi, CCDS, and LGo: investigation. LGi and LGo: writing—original draft. EL, FB, JS, JA, GG-K, LGi, CCDS, and LGo: writing—review. JS and JA: english editing. FB, EL, GG-K, and LGo: funding acquisition. LGo: supervision. All authors contributed to the article and approved the submitted version.

## Conflict of Interest

The authors declare that the research was conducted in the absence of any commercial or financial relationships that could be construed as a potential conflict of interest.

## Supplementary Material

Supplementary Table 1 Bear serum characteristics: Description of different bear serum mix, with single bear ID number, Year of collection, Age, Gender and the protein content of each seasonal mix bear serum. Mean of protein content (mg/ml) ± SD.
