# Supplementary material for: Cardiomyocyte Protection by Hibernating Brown Bear Serum: Toward the Identification of New Protective Molecules Against Myocardial Infarction
Source: Front Cardiovasc Med. 2021 Jul 16;8:687501. doi: 10.3389/fcvm.2021.687501 (PMC8322573; doi:10.3389/fcvm.2021.687501)
Supplement: Supplementary Table 1 — Bear serum characteristics: Description of different bear serum mix, with single bear ID number, Year of collection, Age, Gender and the protein content of each seasonal mix bear serum. Mean of protein content (mg/ml) ± SD. [file Table_1.pdf]

Bear ID_number	Year of collection	Age (year)	Gender	Protein content (mg/ml)	
				Summer	Winter
w1404	2016	3	M	16,17±0,21	16,35±3,36
w1407		3	F		
w1509		2	F		
w1511		2	F		
w1512		2	F		
w1707	2019	3	F	15,95±1,02	18,13±1,29
w1709		3	F		
w1802		2	M		
w1803		2	F		
w1806		2	F		
w1812		2	M		
w1813		2	F		
w1814		2	M		
**N = 13**		**2,31**	**Sex ratio : 4M/9F**		
